# Effects of PEMF and LIPUS Therapy on the Expression of Genes Related to Peripheral Nerve Regeneration in Schwann Cells

**DOI:** 10.3390/ijms252312791

**Published:** 2024-11-28

**Authors:** Mateusz Siwak, Danuta Piotrzkowska, Maciej Skrzypek, Ireneusz Majsterek

**Affiliations:** Department of Clinical Chemistry and Biochemistry, Medical University of Lodz, 92-215 Lodz, Poland; mateusz.siwak@umed.lodz.pl (M.S.); maciej.skrzypek@umed.lodz.pl (M.S.); ireneusz.majsterek@umed.lodz.pl (I.M.)

**Keywords:** peripheral nerve regeneration, pulsed electromagnetic fields (PEMF), low-intensity ultrasound (LIPUS), Schwann cells, gene expression, inflammation

## Abstract

Peripheral nerve regeneration remains a major challenge in neuroscience, despite advancements in understanding its mechanisms. Current treatments, including nerve transplantation and drug therapies, face limitations such as invasiveness and incomplete recovery of nerve function. Physical therapies, like pulsed electromagnetic fields (PEMF) and low-intensity ultrasound (LIPUS), are gaining attention for their potential to enhance regeneration. This study analyzes the effects of PEMF and LIPUS on gene expression in human primary Schwann cells, which are crucial for nerve myelination and repair. Key genes involved in neurotrophin signaling (NGF, BDNF), inflammation (IL-1β, IL-6, IL-10, TNF-α, TGF-β), and regeneration (CRYAB, CSPG, Ki67) were assessed. The results of this study reveal that combined PEMF and LIPUS therapies promote Schwann cell proliferation, reduce inflammation, and improve the regenerative environment, offering potential for optimizing these therapies for clinical use in regenerative medicine.

## 1. Introduction

Peripheral nerve regeneration remains a major challenge in neuroscience. Despite significant advances in understanding the underlying mechanisms, current therapies have notable limitations. Treatments such as autologous nerve grafts, microsurgery, and synthetic conduits are widely used but face challenges. Autologous nerve grafts, considered the “gold standard”, are limited by the availability of donor nerves and potential complications at the donor site. Even after successful grafting, nerve regeneration is often incomplete, leading to sensory and motor deficits. Microsurgery, while precise, is time-consuming, technically demanding, and variable in outcomes, particularly for extensive damage. Synthetic conduits, designed to replace grafts, lack regenerative efficiency and fail to support axonal growth over long distances. Pharmacological and cellular therapies, including stem cells, show potential in preclinical studies but are hindered by delivery difficulties and incomplete understanding of mechanisms [1,2,3].

Given these limitations, there is a growing need for effective, less invasive therapies to promote nerve regeneration. Physical therapies, such as pulsed electromagnetic fields (PEMF) and low-intensity ultrasound (LIPUS), have gained attention for their regenerative potential. These non-invasive approaches stimulate the biological processes involved in tissue repair and regeneration [4]. PEMF therapy exerts profound effects on cellular responses, modulating cell membrane permeability, ion transport, and membrane potential to regulate cellular activity and promote tissue repair [5,6,7,8]. By activating signaling pathways, PEMF stimulates gene transcription, metabolic processes, and homeostasis, supporting functions like muscle contraction and neuronal signaling. It increases levels of protective heat shock proteins (HSPs) and enhances the secretion of growth factors, including BMP-2, BMP-4, IGF, and TGF-β, which are essential for tissue regeneration [9,10,11]. At the tissue level, PEMF reduces inflammation and necrosis through enhanced macrophage activity, while promoting angiogenesis, osteoblast activation, and stem cell proliferation for effective tissue repair [7,8,12]. PEMF has also been shown to relieve pain and inflammation by increasing adenosine receptor expression, stimulating anabolic processes and inhibiting catabolism, and increasing the secretion of growth factors, including BMP-2, BMP-4, and IGF [9,10,13]. These mechanisms contribute to reduced swelling, enhanced immune activity, and improved healing outcomes.

The mechanism of action of LIPUS is complex and involves the induction of micro-bubbles, which stimulate cellular processes such as cell proliferation and differentiation. The potential of LIPUS therapy is based on its ability to stimulate biological processes at the cellular level, such as cell proliferation, synthesis of extracellular matrix proteins, and expression of genes related to tissue healing and regeneration. Studies indicate that LIPUS can accelerate the healing of bone fractures, reduce inflammation, and promote the regeneration of muscle and other soft tissues. The mechanisms of action of this therapy include the activation of signaling pathways such as the MAPK (mitogen-activated protein kinase) pathway, leading to the increased production of growth factors and structural proteins, which are key to tissue regeneration [14,15].

Both PEMF and LIPUS therapies have been explored in various regenerative contexts but remain under-investigated regarding their combined effects on peripheral nerve repair. Schwann cells, key players in peripheral nerve regeneration, are critical targets for these therapies. Schwann cells form myelin sheaths around axons, enabling efficient nerve impulse conduction. Following injury, they enter an activated state, producing growth factors, clearing degraded myelin and guiding regenerating axons through structures known as “Büngner bands” [16]. Schwann cells also modulate inflammation, contributing to a favorable environment for regeneration. Without their activity, nerve repair would be severely impaired, leading to permanent deficits [16,17]. 

This study focused on the expression of the key genes in Schwann cells that regulate myelination and peripheral nerve regeneration. The analyzed genes include NGF (nerve growth factor), BDNF (brain-derived neurotrophic factor), CRYAB (α-crystallin B), CSPGs (chondroitin sulfate proteoglycans), L-1β, IL-6, IL-10, TNF-α, and TGF-β. These genes encode growth factors, heat shock proteins, proteoglycans, and cytokines critical for inflammation regulation, cellular proliferation, differentiation, and creating a regenerative environment. Changes in their expression influence the efficiency of nerve repair. This study assessed whether PEMF and LIPUS therapies modulate their expression, providing insights into the molecular mechanisms behind these therapies. Importantly, this research is among the first to evaluate the simultaneous effects of PEMF and LIPUS on human primary Schwann cells.

Inflammatory cytokines modulate immune responses and nerve repair processes. IL-1β, a pro-inflammatory cytokine, influences Schwann cell functions and nerve regeneration. However, excessive IL-1β expression exacerbates inflammation, impeding repair. Studies suggest controlling IL-1 levels can improve nerve regeneration [1]. Cytokines interact intricately with IL-6 activation, often driven by IL-1β or TNF-α [13]. IL-6 plays a dual role, aiding repair but also contributing to neuropathic pain. Secreted by glial cells and macrophages at injury sites, IL-6 increases inflammation and pain sensitivity by modulating neuronal activity. Blocking the IL-6 pathway may offer therapeutic benefits for reducing pain and enhancing repair [18,19]. TNF-α regulates immune responses and inflammation, influencing neuropathic pain and nerve repair. It activates neurons and recruits pro-inflammatory immune cells like M1 macrophages and neutrophils, indirectly affecting nociception and regeneration [2,20,21,22]. In contrast, IL-10, an anti-inflammatory cytokine, minimizes tissue damage and fosters repair by suppressing pro-inflammatory cytokines like TNF-α. Elevated IL-10 levels in injured nerve tissues create an environment conducive to regeneration, making it a therapeutic target [23,24]. TGF-β, another crucial cytokine, balances inflammation and promotes axonal regeneration and remyelination, essential for restoring nerve function. It modulates the immune response, preventing excessive inflammation while enhancing repair. Studies suggest that PEMF may regulate miRNA-21, increasing TGF-β expression and further promoting regeneration [1,25,26,27]. Neurotrophins such as NGF and BDNF are vital for peripheral nerve repair. BDNF supports neuronal survival, remyelination, and synaptic plasticity, all critical for functional recovery. It also regulates other neurotrophic factors, making it a promising target for therapies to treat neuropathies [28,29]. NGF, essential for sensory neuron growth and differentiation, plays a central role in axonal regeneration and neuropathic pain modulation. It regulates pain receptor expression and accelerates nerve healing, highlighting its potential in both regenerative and pain therapies [28,29,30]. CSPGs inhibit axonal growth by forming glial scars and reducing growth factor availability at injury sites. Strategies to neutralize or modify CSPGs could improve regeneration outcomes [31,32]. Conversely, CRYAB, a heat shock protein, protects nerve cells from oxidative stress and promotes remyelination. By stabilizing cytoskeletal structures and preventing protein aggregation, CRYAB enhances nerve repair and serves as a promising therapeutic target [33,34]. Ki-67, a proliferation marker, is associated with Schwann cell activity after nerve injury. Its increased expression signals active repair processes such as remyelination and axonal support. Tracking Ki-67 levels provides insights into the progression of regeneration and therapy effectiveness [34,35,36,37].

This study emphasizes the importance of cytokines, neurotrophins, and structural proteins in nerve regeneration and explores how PEMF and LIPUS therapies modulate these key factors to promote repair and functional recovery.

In summary, the aim of our study was to investigate the effects of PEMF (pulsed electromagnetic field) and LIPUS (low-intensity ultrasound) therapies on the expression of key genes in Schwann cells, which play a fundamental role in peripheral nerve regeneration. We want to investigate how these therapies can modulate the expression of genes related to repair processes, such as NGF, BDNF, CRYAB, IL-10, TGF-β, and pro-inflammatory and anti-inflammatory cytokines, including IL-1β, IL-6, TNF-α, and CSPGs. Our research aims to better understand the molecular mechanisms that may promote nerve regeneration and contribute to the development of new, more effective therapeutic strategies. The results of this project may provide key insights not only into the potential of PEMF and LIPUS in regenerative medicine, but also open new perspectives in the treatment of peripheral nerve injury.

## 2. Results

The effects of PEMF and LIPUS fields at specific frequencies on Schwann cell cultures were assessed (PEMF 100 Hz or 1000 Hz, LIPUS at 20 kHz or 40 kHz, and, after combined therapy, PEMF 100 Hz with LIPUS 20 kHz, PEMF 1000 Hz with LIPUS 20 kHz, PEMF 100 Hz with LIPUS 40 kHz, PEMF 1000 Hz with LIPUS 40 kHz). Gene expression analysis was performed for 11 genes, including the endogenous control gene GAPDH and 10 test genes, namely NGF (nerve growth factor), BDNF (brain-derived neurotrophic factor), CRYAB (α-crystallin B), CSPGs (chondroitin sulfate proteoglycans), IL-1β (interleukin-1β), IL-6 (interleukin-6), IL-10 (interleukin-10), TNF-α (tumor necrosis factor-α), TGF-β (transforming growth factor-beta), and MKI67 (Ki-67 marker of proliferation). Statistical analysis showed that the expression of genes related to inflammation, pro-inflammatory cytokines (IL-1β, IL-6, TNF-α), and inhibitors of axonal growth (CSPG) were reduced in PEMF- and/or LIPUS-treated Schwann cells compared to control cells.

In contrast, genes that play a key role in the regulation of anti-inflammatory processes (IL-10, TGF-β), cell proliferation (KI67), and the creation of an environment conducive to axonal regeneration (BDNF, NGF, CRYAB) were significantly increased.

### 2.1. Analysis of the Expression of Pro-Inflammatory and Anti-Inflammatory Cytokines IL-1β, IL-6, TNF-α, IL-10, and TGF-β in Schwann Cells Treated with PEMF and/or LIPUS

**Figure 1:**
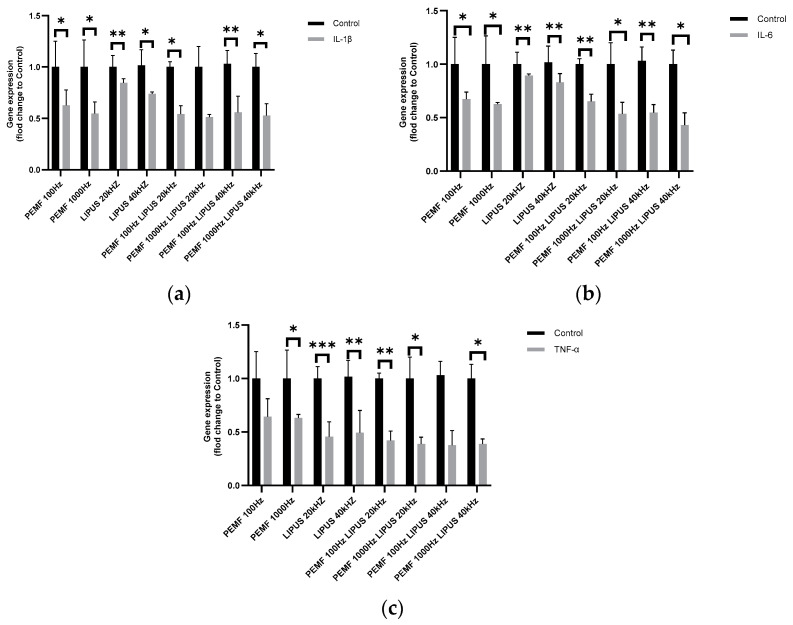
Gene expression of pro-inflammatory cytokines (**a**) IL-1; (**b**) IL-6; (**c**) TNF-α in Schwann cells after treatment with PEMF at 100 Hz or 1000 Hz and LIPUS at 20 kHz or 40 kHz, and after combined therapy with PEMF 100 Hz and LIPUS 20 kHz, PEMF 1000 Hz and LIPUS 20 kHz, PEMF 100 Hz and LIPUS 40 kHz, and PEMF 1000 Hz and LIPUS 40 kHz, as well as in control cells. Groups of data were compared using the Mann–Whitney U test (* indicates statistical significance at *p* < 0.05, ** indicates statistical significance at *p* < 0.01, *** indicates statistical significance *p* < 0.001). Data are presented as mean ± SEM).

The exposure of Schwann cells to combined PEMF 100 Hz or 1000 Hz with LIPUS at 20 kHz or 40 kHz resulted in an approximately 50% reduction in IL-1β expression compared to control (Figure 1a), with statistical significance for PEMF 100 Hz with 20 kHz at *p* < 0.05, PEMF 100 Hz with LIPUS 40 kHz at *p* < 0.01, and PEMF 1000 Hz and LIPUS 40 kHz at *p* < 0.05. In contrast, LIPUS 20 kHz and 40 kHz field therapy alone reduced IL-1β expression in Schwann cells by approximately 20% and 30%, respectively, compared to control, with statistical significances of *p* < 0.01 and *p* < 0.05, respectively (Figure 1a). PEMF field therapy at 100 Hz and 1000 Hz reduced IL-1β expression by approximately 50% and 40%, respectively, compared to control cells, with statistical significances of *p* < 0.05 and *p* < 0.05, respectively (Figure 1a).

The greatest reduction in IL-6 expression, of 60%, was observed in Schwann cells after combined treatment with PEMF 1000 Hz with LIPUS 40 kHz (*p* < 0.05) (Figure 1b). After the exposure of Schwann cells to a 1000 Hz PEMF field, in combination with LIPUS 20 kHz or PEMF 100 Hz with LIPUS 40 kHz, IL-6 expression decreased by 50%, with statistical significances of *p* < 0.05 and *p* < 0.01 (Figure 1b). When Schwann cells were exposed to a 20 kHz LIPUS field and 100 Hz PEMF, IL-6 level decreased by 40%, with a statistical significance of *p* < 0.01(Figure 1b). Application of the 20 kHz and 40 kHz LIPUS field alone reduced IL-6 expression in Schwann cells by approximately 10% and 20%, respectively, compared to control cells, with statistical significance *p* < 0.05 and *p* < 0.001, respectively (Figure 1b). 

In contrast, a PEMF field at 100 Hz and 1000 Hz reduced IL-6 expression in Schwann cells by approximately 40%, with statistical significances of *p* < 0.05 and *p* < 0.0001, respectively (Figure 1b). Compared to controls, the greatest reduction in TNF-α expression (60%) in Schwann cells was observed following combination therapy with PEMF with 100 Hz or 1000 Hz, along with LIPUS at 20 kHz or 40 kHz, with statistical significance for the combination of PEMF 100 Hz with LIPUS 20 kHz at *p* < 0.05, PEMF 1000 Hz with LIPUS 20 kHz at *p* < 0.05, and both combinations of PEMF 100 Hz and PEMF 1000 Hz with LIPUS 40 kHz at *p* < 0.05 (Figure 1c). Compared to the control, a slightly weaker effect in TNF-α expression, an approximately 55% reduction, was observed after exposing Schwann cells to a 20 kHz or 40 kHz LIPUS field, with statistical significance of *p* < 0.001 and *p* < 0.01, respectively (Figure 1c).

The expression level of IL-10 in Schwann cells after exposure to LIPUS and PEMF fields increased at all frequencies tested. When Schwann cells were exposed to a 100 Hz or 1000 Hz PEMF field alone, TNF-α expression increased by 40% (no statistical significance was shown for 100 Hz and 1000 Hz PEMF) (Figure 1a).

**Figure 2:**
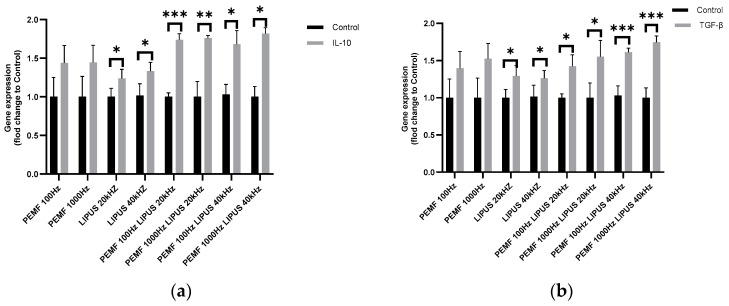
Gene expression of anti-inflammatory cytokines (**a**) IL-10; (**b**) TGF-β in Schwann cells after treatment with PEMF 100 Hz or 1000 Hz and LIPUS 20 kHz or 40 kHz, and after combined therapy with PEMF 100 Hz with LIPUS 20 kHz, PEMF 1000 Hz with LIPUS 20 kHz, PEMF 100 Hz with LIPUS 40 kHz, and PEMF 1000 Hz and LIPUS 40 kHz, as well as in control cells. Groups of data were compared using the Mann–Whitney U test (* indicates statistical significance at *p* < 0.05, ** indicates statistical significance at *p* < 0.01, *** indicates statistical significance *p* < 0.001). Data are presented as mean ± SEM).

The highest increase in IL-10 expression, of approximately 70%, was observed in Schwann cells after the combined therapy of LIPUS 20 kHz or 40 kHz with PEMF 100 Hz or 1000 Hz, with statistical significance for PEMF 100 Hz with LIPUS 20 kHz at *p* < 0.001, PEMF 1000 Hz with LIPUS 20 kHz at *p* < 0.01, PEMF 100 Hz with LIPUS 40 kHz at *p* < 0.05, and PEMF 1000 Hz with LIPUS 40 kHz at *p* < 0.05 (Figure 2a). Application of the 20 kHz and 40 kHz LIPUS field alone resulted in a 25–30% increase in IL-10 expression in Schwann cells compared to control cells, with a statistical significance of *p* < 0.05 in both cases (Figure 2a). The 100 Hz and 1000 Hz PEMF field induced an approximately 40% increase in IL-10 expression in Schwann cells (no statistical significance was obtained) (Figure 2a). 

TGF-β gene expression in Schwann cells was up-regulated after the exposure of Schwann cells to LIPUS and/or PEMF fields at all frequencies tested. The highest increase in TGF-β expression was observed after the combined treatment of LIPUS 40 kHz with PEMF 1000 Hz, amounting to 70% (*p* < 0.001), and after the treatment of LIPUS 40 kHz with PEMF 100 Hz, amounting to approximately 60% (*p* < 0.001) (Figure 2b). Cells exposed to 100 Hz or 1000 Hz PEMF fields with 20 kHz LIPIS resulted in increased TGF-β expressions of 40% (*p* < 0.05) and 55% (*p* < 0.05), respectively (Figure 2b). Exposure of Schwann cells to a 100 Hz or 1000 Hz PEMF field increased TGF-β expression by 40% and 50%, respectively, but did not show statistical significance. In contrast, a LIPUS field of 20 kHz and 40 kHz alone increased TGF-β expression in Schwann cells by 25% (*p* < 0.05) and 30% (*p* < 0.05), respectively (Figure 2b).

### 2.2. Analysis of Neurotrophin (BDNF and NGF) Expression in Schwann Cells Given PEMF and/or LIPUS Therapy

**Figure 3:**
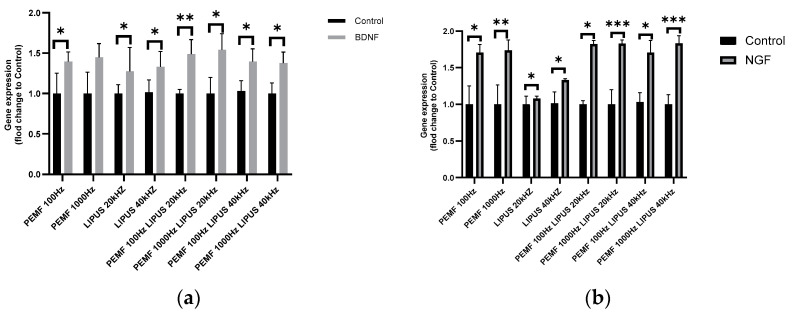
Gene expression of neurotrophins (**a**) BDNF; (**b**) NGF in Schwann cells after treatment with PEMF 100 Hz or 1000 Hz and LIPUS 20 kHz or 40 kHz, and after combined therapy with PEMF 100 Hz and LIPUS 20 kHz, PEMF 1000 Hz and LIPUS 20 kHz, PEMF 100 Hz and LIPUS 40 kHz, and PEMF 1000 Hz and LIPUS 40 kHz, as well as in control cells. Groups of data were compared using the Mann–Whitney U test (* indicates statistical significance at *p* < 0.05, ** indicates statistical significance at *p* < 0.01, *** indicates statistical significance *p* < 0.001). Data are presented as mean ± SEM).

The highest increase in BDNF gene expression, of 50%, was observed after the exposure of Schwann cells to a 1000 Hz PEMF field in combination with 20 kHz LIPUS and a 100 Hz PEMF field in combination with 20 kHz LIPUS, with statistical significances of *p* < 0.01 and *p* < 0.05, respectively (Figure 3a). In contrast, the exposure of Schwann cells to PEMF 1000 Hz with LIPUS 40 kHz and PEMF 100 Hz with LIPUS 40 kHz resulted in an approximately 40% increase in BDNF expression, with statistical significances of *p* < 0.05 and *p* < 0.05, respectively (Figure 3a).

The exposure of Schwann cells to a 100 Hz or 1000 Hz PEMF field alone resulted in a 40% increase in BDNF expression, with statistical significance for 100 Hz PEMF at *p* < 0.05 and no statistical significance obtained for PEMF. In contrast, the LIPUS field at 40 kHz and 20 kHz resulted in an increase in BDNF expression of approximately 25% and 30%, respectively, with statistical significances of *p* < 0.05 and *p* < 0.05, respectively (Figure 3a). 

Combination therapy with LIPUS and PEMF resulted in an over 80% increase in NGF expression in Schwann cells, with statistical significance for PEMF 100 Hz and LIPUS 20 kHz at *p* < 0.05, PEMF 1000 Hz and LIPUS 20 kHz at *p* < 0.001, PEMF 100 Hz and LIPUS 40 kHz at *p* < 0.05, and PEMF 1000 Hz and LIPUS 40 kHz at *p* < 0.001 (Figure 3b).

Schwann cells exposed to a 100 Hz and 1000 Hz PEMF field alone showed a 70% and 80% increase in NGF expression, respectively, with statistical significances of *p* < 0.05 and *p* < 0.01, respectively. In contrast, a 20 kHz and 40 kHz LIPUS field alone resulted in an approximately 20% increase in NGF expression, with statistical significances of *p* < 0.001 for both frequencies (Figure 3b).

### 2.3. Analysis of the Expression of the Axon Growth Inhibitor CSPG in Schwann Cells Under PEMF and/or LIPUS Therapy

**Figure 4:**
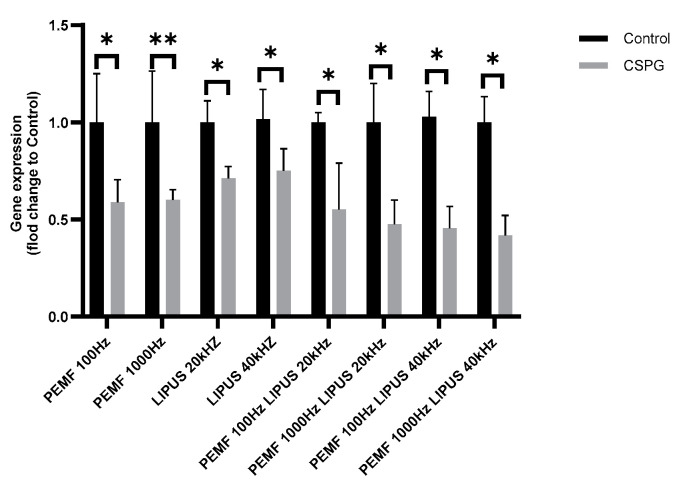
CSPG gene expression in Schwann cells after treatment with PEMF 100 Hz or 1000 Hz and LIPUS 20 kHz or 40 kHz, and after combined therapy with PEMF 100 Hz and LIPUS 20 kHz, PEMF 1000 Hz and LIPUS 20 kHz, PEMF 100 Hz and LIPUS 40 kHz, and PEMF 1000 Hz and LIPUS 40 kHz, as well as in control cells. Groups of data were compared using the Mann–Whitney U test (* indicates statistical significance at *p* < 0.05, ** indicates statistical significance at *p* < 0.01). Data are presented as mean ± SEM).

CSPG gene expression in Schwann cells decreased after LIPUS and PEMF treatment at all frequencies tested. The greatest reduction in CSPG gene expression, of more than 50%, was observed in Schwann cells exposed to a LIPUS field of 40 kHz, PEMF field of 100 Hz or 1000 Hz, PEMF field of 1000 Hz, and a LIPUS field of 20 kHz, with a statistical significance for all cases of *p* < 0.05. A PEMF field of 100 Hz and a LIPUS field of 20 kHz resulted in a 40% reduction in CSPG expression in Schwann cells (*p* < 0.05) (Figure 4). The 20 kHz and 40 kHz LIPUS field alone caused an approximately 30% reduction in CSPG expression (*p* < 0.05). In contrast, the PEMF field at frequencies of 100 Hz and 1000 Hz decreased CSPG expression in Schwann cells by approximately 40% compared to control cells, with statistical significances of *p* < 0.05 and *p* < 0.01, respectively (Figure 4).

**Figure 5:**
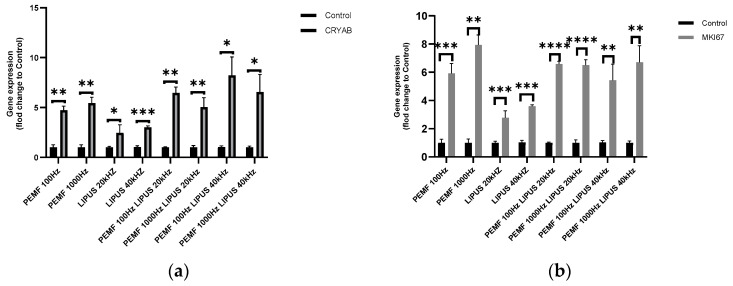
Expression of (**a**) CRYAB; and (**b**) MKI67 in Schwann cells after treatment with PEMF 100 Hz or 1000 Hz and LIPUS 20 kHz or 40 kHz, and after combined treatment with PEMF 100 Hz and LIPUS 20 kHz, PEMF 1000 Hz and LIPUS 20 kHz, PEMF 100 Hz and LIPUS 40 kHz, and PEMF 1000 Hz and LIPUS 40 kHz, as well as in control cells. Groups of data were compared using the Mann–Whitney U test (* indicates statistical significance at *p* < 0.05, ** indicates statistical significance at *p* < 0.01, *** indicates statistical significance *p* < 0.001, **** indicates statistical significance *p* < 0.0001). Data are presented as mean ± SEM).

In Schwann cells treated with a 1000 Hz or 100 Hz PEMF field with 40 kHz LIPUS, the highest increase in CRYAB gene expression, of 800% (*p* < 0.05) and 700% (*p* < 0.05), respectively, was observed. A 100 Hz or 1000 Hz PEMF field combined with 20 kHz LIPUS resulted in an increase in CRYAB expression of approximately 650% (*p* < 0.01) and 550% (*p* < 0.01), respectively. Exposure of Schwann cells to a 100 Hz or 1000 Hz PEMF field alone induced a 500% (*p* < 0.01) and 550% (*p* < 0.01) increase in CRYAB expression, respectively, compared to control cells. In contrast, a 20 kHz and 40 kHz LIPUS field alone induced a 200% and 300% increase in CRYAB expression, respectively, with statistical significances of *p* < 0.05 and *p* < 0.001 (Figure 5a). 

Schwann cells exposed to PEMF and LIPUS fields at all frequencies tested showed a significant increase in MKI67 expression. The highest increase in MKI67 expression in Schwann cells, of 700%, was observed with a 1000 Hz PEMF field combined with 40 kHz LIPUS (*p* < 0.01), as well as with a 1000 Hz PEMF field alone (*p* < 0.001) (Figure 5a). 

Combination therapy of PEMF 100 Hz with LIPUS 20 kHz (*p* < 0.05) and PEMF 1000 Hz with LIPUS 20 kHz (*p* < 0.0001) resulted in a more than 650% increase in MKI67 expression in Schwann cells. In contrast, PEMF treatment of 100 Hz with LIPUS 40 kHz (*p* < 0.01) led to a 550% increase in MKI67 expression compared to control cells (Figure 5a).

Application of 100 Hz PEMF alone induced a 600% increase in Ki67 expression at a statistical significance of *p* < 0.001. In contrast, a 20 kHz or 40 kHz LIPUS field induced an approximately 300% increase in MKI67 expression in Schwann cells, also at a stastitical significance of *p* < 0.001(Figure 5a).

### 2.4. Analysis of Genotoxicity of Schwann Cells in PEMF and LIPUS Field

**Figure 6:**
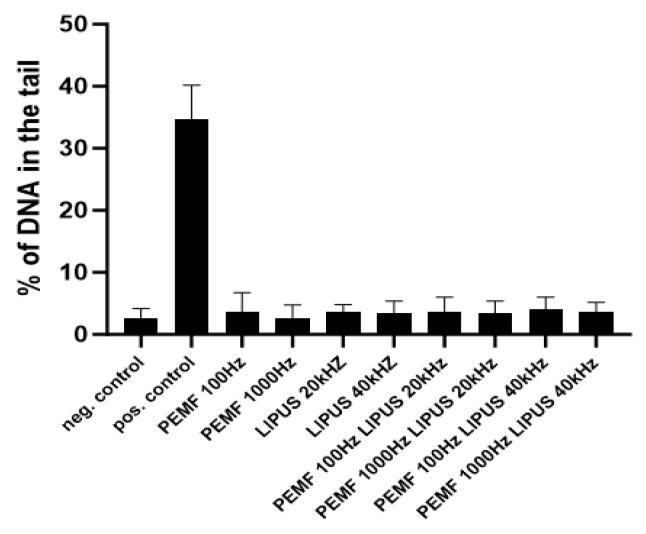
Analysis of genotoxicity in Schwann cells after treatment with PEMF 100 Hz or 1000 Hz and LIPUS 20 kHz or 40 kHz, and after combined treatment with PEMF 100 Hz and LIPUS 20 kHz, PEMF 1000 Hz and LIPUS 20 kHz, PEMF 100 Hz and LIPUS 40 kHz, and PEMF 1000 Hz and LIPUS 40 kHz, as well as in control cells. The analysis was performed using the alkaline version of the comet assay after 10 days of PEMF and/or LIPUS field therapy. Cells suspended in 10% DMSO were used as a positive control. Cells suspended in 1.5 mL of complete culture medium were used as a negative control. 1–40 kHz LIPUS + PEMF 4 Hz.

Genotoxicity analysis, performed on Schwann cells using an alkaline version of the comet assay after 10-day treatment with LIPUS (20 kHz or 40 kHz) and/or PEMF at 100 or 1000 Hz, showed no significant DNA damage. The level of DNA damage in the samples tested was comparable to the negative control, being approximately 5% in all cases. The positive control was Schwann cells treated with 10% DMSO, in which more than 30% DNA damage was observed (Figure 6).

### 2.5. Analysis of Cytotoxicity of Schwann Cells in PEMF and LIPUS Field

**Figure 7:**
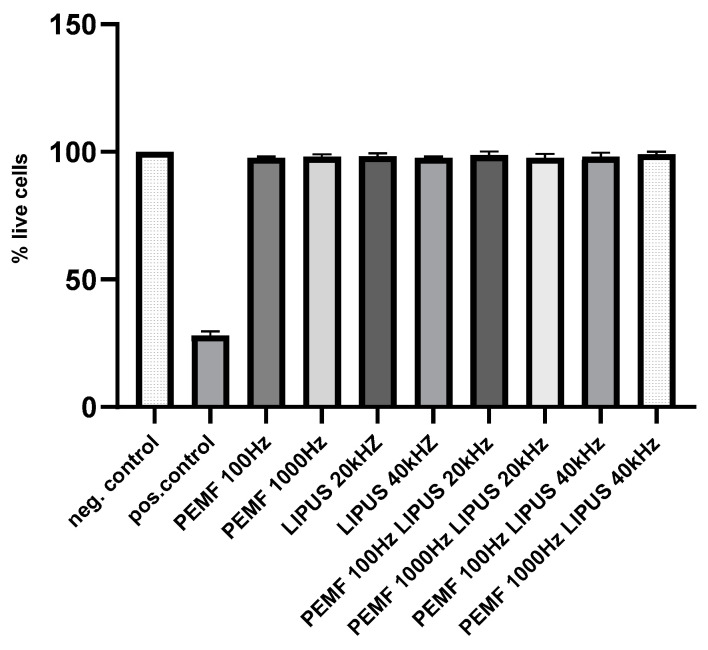
Analysis of cytotoxicity (XTT test) f in Schwann cells after treatment with PEMF 100 Hz or 1000 Hz and LIPUS 20 kHz or 40 kHz, and after combined treatment with PEMF 100 Hz and LIPUS 20 kHz, PEMF 1000 Hz and LIPUS 20 kHz, PEMF 100 Hz and LIPUS 40 kHz, and PEMF 1000 Hz and LIPUS 40 kHz, as well as in control cells. Cells treated with 10% DMSO (dimethyl sulfoxide) were used as positive control. Cells cultured in standard medium served as negative control.

The obtained results indicate that none of the applied PEMF and LIPUS therapies, nor the combined therapy, showed a significant cytotoxic effect on Schwann cells. The number of live cells was comparable to the control group, in which cells were grown under standard conditions. This means that both PEMF and LIPUS fields, as well as their combined use, are safe for the tested cell line and do not negatively affect its basic life functions (Figure 7).

## 3. Discussion

Schwann cells are pivotal during peripheral nerve regeneration, performing numerous supportive functions essential for the survival of damaged axons, their regeneration, and the re-innervation of injured tissue. In damaged nerves, an increased expression of trophic factors prevents neuronal death, while cytokines facilitate macrophage recruitment. These processes also involve autophagy of residual myelin, the formation of regenerative pathways for axons, and eventual remyelination [38].

Studies indicate that pulsed electromagnetic field (PEMF) therapy accelerates Schwann cell proliferation and differentiation, which are critical for nerve regeneration and repair [39]. PEMF also promotes neuron growth and provides protective effects on neural stem cells under hypoxic conditions, underscoring its potential therapeutic benefits [40]. Low-intensity pulsed ultrasound (LIPUS) enhances Schwann cell migration and viability and stimulates their proliferation and migration in vitro [41]. LIPUS further influences gene expression related to mesenchymal cell differentiation, emphasizing its role in cell differentiation [39,40,41].

Comparing the effects of PEMF and LIPUS on Schwann cell function reveals that they operate through distinct mechanisms. While PEMF predominantly fosters cell proliferation and differentiation, promoting nerve regeneration, LIPUS primarily enhances cell migration and viability, which are crucial for peripheral nerve healing. Both therapies show promise in improving Schwann cell function but differ in their mechanisms of action and target effects, broadening their therapeutic applications in regenerative medicine and tissue engineering [39,41].

In this study, we examined the expression of genes crucial for Schwann-cell-mediated myelination and peripheral nerve regeneration. We analyzed genes encoding growth factors, heat shock proteins, proteoglycans, and cytokines, including NGF, BDNF, CRYAB, CSPGs, MKI67, IL-1β, IL-6, IL-10, TNF-α, and TGF-β. These genes regulate inflammation and cell proliferation and differentiation, creating an environment conducive to axonal regeneration. Given their significance, we explored whether PEMF and LIPUS could modulate their expression in Schwann cells to gain insights into the molecular mechanisms underlying these therapies.

Pro-inflammatory cytokines play a crucial role in modulating the immune response and repairing damaged peripheral nerves. Both PEMF and LIPUS therapies modulate the expression of pro-inflammatory cytokines (IL-1β, IL-6, TNF-α) and anti-inflammatory cytokines (IL-10, TGF-β) [42,43]. These cytokines interact complexly, influencing each other’s expression and function. For instance, IL-6 activation by IL-1β or TNF-α occurs via the p38 MAPK pathway, which is also involved in TGF-β-induced gene expression [44].

Modulating cytokine expression through PEMF and LIPUS therapies may regulate the inflammatory response, creating a more conducive environment for peripheral nerve regeneration. Our results for pro-inflammatory cytokines (TNF-α, IL-1β, IL-6; Figure 1a–c) align with previous studies demonstrating the anti-inflammatory effects of LIPUS and PEMF across various cell models [45,46]. Concurrently, we confirmed an increase in anti-inflammatory cytokines (IL-10, TGF-β; Figure 2a,b), consistent with previous reports [42]. Fontana’s work has shown that PEMF therapy significantly decreases TNF-α and IL-6 levels in inflammatory models [47]. In vivo studies further support these findings. For example, Ito demonstrated that LIPUS therapy reduced TNF-α and IL-6 expression seven days after nerve injury in rats [48]. TNF-α is pivotal in the inflammatory process that can lead to peripheral nerve damage. Elevated TNF-α levels in nerve tissues are often linked with increased neuropathic pain, as this cytokine promotes demyelination and axonal degeneration, impairing nerve conduction. Preclinical studies suggest that blocking TNF-α may offer therapeutic potential in mitigating peripheral nerve damage. Our findings show that both LIPUS and PEMF therapies significantly impact TNF-α expression in Schwann cells, which is consistent with existing literature [39,42]. Specifically, LIPUS and PEMF have the potential to reduce pro-inflammatory markers like TNF-α, which is crucial for promoting peripheral nerve regeneration. Ross has demonstrated that PEMF significantly reduces TNF-α levels in nerve tissues, leading to decreased inflammation and improved peripheral nerve regeneration post-injury [42]. Similarly, Acheta has suggested that LIPUS promotes nerve regeneration by reducing TNF-α and other inflammatory markers [39].

Our results indicate a synergistic effect when combining LIPUS and PEMF therapies, leading to a greater reduction in TNF-α levels (Figure 1c). This is the first study suggesting that the combination of these therapies may be more effective than either therapy alone. Notably, the reduction in TNF-α expression observed with either therapy also independently confirms that their effects are more pronounced when used together (Figure 1c).

Interleukin-10 (IL-10), a key immune response regulator with potent anti-inflammatory properties, plays a vital role in peripheral nerve regeneration. IL-10 inhibits proinflammatory cytokines (TNF-α, NF-κB) and maintains tissue homeostasis [49,50]. By curbing excessive inflammation, IL-10 protects nervous tissue from secondary damage and encourages repair processes, making it a promising target for nerve regeneration therapies [24,51,52].

Our analysis revealed a significant increase in IL-10 expression following LIPUS and PEMF therapy, particularly in Schwann cells subjected to combined therapy or LIPUS alone (Figure 2a). Modulating the IL-10 pathway through these therapies offers a potential strategy for reducing chronic pain, limiting scarring, and accelerating nerve regeneration. IL-10 also interacts with TGF-β, with both cytokines synergistically promoting anti-inflammatory responses and supporting regenerative processes.

TGF-β plays a dual role in regulating inflammation and promoting tissue regeneration. In Schwann cells, TGF-β modulates immune responses and facilitates axonal regeneration and remyelination. The increased expression of TGF-β observed with LIPUS and PEMF therapies suggests these treatments may enhance nerve regeneration by reducing inflammation and supporting tissue repair [25,53].

Our findings on BDNF and NGF expression align with existing literature highlighting the neuroprotective and regenerative roles of these neurotrophic factors. Ren demonstrated that LIPUS therapy significantly increased BDNF and NGF levels, supporting Schwann cell viability and proliferation [54]. Similarly, our study showed a significant increase in neurotrophin gene expression (BDNF, NGF) following LIPUS exposure (Figure 3a,b). Wang has also reported that PEMF therapy has neuroprotective effects, significantly increasing BDNF expression, which we corroborated in our study (Figure 3a) [54]. Additionally, Shao has confirmed that PEMF could enhance neurotrophin levels, promoting nerve regeneration [55]. Notably, the greatest increase in BDNF and NGF expression was observed with concurrent stimulation by LIPUS at 20 kHz and PEMF at 1000 Hz, underscoring the potential of these fields to stimulate regenerative processes (Figure 3a,b).

CRYAB, a small heat shock protein, plays a critical role in protecting nerve cells from oxidative stress and preventing protein aggregation. CRYAB is believed to inhibit harmful pro-inflammatory responses by macrophages during the later stages of peripheral nerve regeneration [56]. Previous studies indicate that CRYAB can reduce the secretion of pro-inflammatory cytokines, including IL-6, IL-1β, IL-12, and TNF-α [33]. Although there are limited studies on the synergistic effects of LIPUS and PEMF on Schwann cells, our findings suggest that this combination may be more effective in supporting peripheral nerve regeneration. We observed a significant increase in CRYAB expression following combined LIPUS and PEMF therapy. This is consistent with Hannemann’s work, which has shown that these therapies modulate CRYAB expression related to stress responses and cell protection [57]. LIPUS combined with PEMF also induced a notable increase in CRYAB expression. These findings align with studies suggesting that lower LIPUS frequencies, when combined with PEMF, can effectively modulate gene expression [34,57,58].

Chondroitin sulfate proteoglycans (CSPGs) are key extracellular matrix components that hinder axonal regeneration in damaged peripheral nerves. CSPGs inhibit axon growth and remyelination, primarily by interacting with growth-inhibitory receptors on regenerating axons [59,60,61]. However, in this study, neither LIPUS nor PEMF significantly reduced CSPG expression, which remained consistent across all treatment groups (Figure 4). This finding indicates that the therapeutic effects of LIPUS and PEMF may be mediated through other pathways, such as the modulation of cytokine expression, rather than by directly affecting CSPGs.

The proliferation marker MKI67 is essential for Schwann cell proliferation during peripheral nerve regeneration [37]. Our results revealed a significant increase in MKI67 expression following LIPUS and PEMF therapy (Figure 5b), suggesting that these therapies may enhance Schwann cell proliferation and contribute to nerve regeneration. Balakrishnan has demonstrated that LIPUS promotes Schwann cell proliferation and migration, consistent with our findings [41]. Additionally, Acheta has shown that PEMF therapy enhances Schwann cell proliferation, further supporting our results [39].

Our investigation also focused on assessing the cytotoxicity and genotoxicity of PEMF and LIPUS therapies in Schwann cells to ensure the safety of these treatments.

Although some studies suggest that ultrasound (LIPUS) exposure may lead to DNA damage, our research did not demonstrate a genotoxic effect from PEMF and LIPUS therapies on Schwann cells, as confirmed by the comet assay (Figure 6) [62]. 

The results demonstrated that neither PEMF nor LIPUS, applied individually or in combination, induced significant cytotoxic or genotoxic effects. Cytotoxicity evaluation revealed comparable cell viability between treated and control groups, with no discernible adverse effects on cell proliferation. Furthermore, genotoxicity analysis using the comet assay confirmed the absence of DNA damage, as evidenced by low levels of DNA strand breaks similar to the negative control.

These findings indicate that PEMF and LIPUS therapies are safe for Schwann cells, preserving their structural and functional integrity and supporting their potential clinical application in peripheral nerve regeneration. These findings align with previous studies that also did not show DNA damage after these therapies. Our findings demonstrated how these therapies modulate gene expression in Schwann cells, which could be crucial for their potential clinical applications. Future research should focus on optimizing treatment parameters, such as frequency and duration, to fully leverage their regenerative potential. Further in vivo studies and clinical trials are essential in order to translate these findings into effective therapeutic strategies for patients with peripheral nerve injuries. It should be emphasized that this study is a starting point for further research. In the future, we plan to conduct a more comprehensive analysis of the gene expression profile using techniques such as microarrays or RNA sequencing to obtain a more complete picture of the molecular mechanisms underlying the observed changes.

## 4. Materials and Methods

### 4.1. Preparation of the Human Schwann Cells Cell Line

All in vitro analyses were performed in an experimental model using a commercially available Human Schwann Cells line (Cat.1701, ScienCell, Carlsbad, CA, USA). Cells were cultured in Schwann Cell Medium (Cat.1701, ScienCell), FBS (ScienCell, Cat.0025), Schwann Cell Growth Supplement (Cat.1752, ScienCell), and 1% penicillin/streptomycin solution (P/S, Cat.0503, ScienCell). Prior to culture establishment, plates were coated with Poly-L-Lysine (A3890401, Thermo Fisher Scientific). Cells were cultured under standard conditions (37 °C; 5% CO_2_; 95% humidity) according to the manufacturer’s guidelines.

### 4.2. Exposure of Schwann Cells in PEMF and/or LIPUS Field

Schwann cells were seeded into 12-well plates (2500 per well) and then cultured for 10 days in Schwann Cell Medium (Cat.1701, ScienCell) for 40 cycles/40 s, with a 10 s break between cycles. Every 24 h, cells were exposed to LIPUS and/or PEMF fields. At the same time, each time before and after PEMF and/or LIPUS exposure, cells were analyzed under a microscope to assess cell viability (percentage of cell confluence and adhesion). The apparatus that was used to generate the LUPUS and PEMF field was located in an incubator to ensure ideal cell culture conditions (5% CO_2_, humidity and 37 °C). After exposure to a given LIPUS/PEMF field, plates with cells were transferred to a culture incubator without the LIPUS and PEMF fields. Schwann cells were exposed to different combinations of LUPUS and PEMF fields.

### 4.3. Therapeutic Programs of PEMF and LIPUS Used

Schwann cells were given PEMF and/or LIPUS therapy: 1.PEMF 100 Hz/20 s/, pause—20 s; loop 20 times; 2. PEMF 1000 Hz/20 s/, pause—20 s; loop 20 times; 3.LIPUS 20 kHz/20 s/, pause—20 s; loop 20 times; 4.LIPUS 40 kHz/20 s/, pause—20 s; loop 20 times; 5. LIPUS 20 kHz/20 s/ + PEMF 100 Hz/20 s/, pause—20 s; loop 20 times; 6. LIPUS 20 kHz/20 s/ + PEMF 1000 Hz/20 s/, pause—20 s; loop 20 times; 7. LIPUS 40 kHz/20 s/ + PEMF 100 Hz/20 s/, pause—20 s; loop 20 times; 8. LIPUS 40 kHz/20 s/ + PEMF 1000 Hz/20 s/, pause—20 s; loop 20 times.

### 4.4. RNA Extraction

Total RNA was isolated from Schwann cells exposed to electromagnetic field as follows: PEMF (100 or 1000 Hz), LIPUS (20 or 40 Hz), PEMF (100 Hz) combined with LIPUS (20 Hz), PEMF (1000 Hz) combined with LIPUS (20 Hz), PEMF (100 Hz) combined with LIPUS (40 Hz), PEMF (1000 Hz) combined with LIPUS (40 Hz), all using the RNeasy Mini Kit (Qiagen, Hilden, Germany) according to the protocol’s instructions. The concentration and quality of the RNA that was extracted was determined by measuring the samples at 260 and 280 nm using a Multiskan Sky-high Microplate spectrophotometer (Thermo Scientific, Thermo Fisher Scientific, Waltham, MA, USA).

### 4.5. Generation of Single-Stranded cDNA 

The obtained RNA was used to convert 100 ng of total RNA to cDNA in a single 10 μL reaction using the High-Capacity cDNA Reverse Transcription Kit (Applied Biosystems, Thermo Fisher Scientific, Waltham, MA, USA), according to the manufacturer’s instructions. To perform reverse transcription, a thermal cycler with a VeritiPro name (Applied Biosystems™, Thermo Fisher Scientific, Waltham, MA, USA) was employed according to the manufacturer’s recommendations.

### 4.6. Real-Time Quantitative PCR (qPCR)

The expression of genes in the experimental and control groups was evaluated using Taqman technology. For qPCR, 10 ng of the generated cDNA was analyzed using the CFX connect Real-Time PCR system (Bio-Rad, Hercules, CA, USA). The cDNA was subjected to quantitative real-time PCR using the CFX Connect Real-Time System (BioRad, Hercules, CA, USA) with TaqMan PCR Master Mix and TaqMan Gene Expression Assays (Applied Biosystems, Waltham, MA, USA) for IL-1β, IL-6, TNF-α, IL-10, TGF-β, BDNF, NGF, CSPG, CRYAB, Ki67, GAPDH mRNA (IL-1β Hs02166755_m1, IL-6 Hs00174131_m1, TNF-α Hs00174128_m1, IL-10 Hs00961622_m1, TGF-β Hs00998133_m1, BDNF Hs02718934_s1, NGF Hs00171458_m1, CSPG Hs00426981_m1, CRYAB Hs00157107_m1, Ki67 Hs00606991_m1, and GAPDH Hs03929097_g1), which were used according to the manufacturer’s instruction. The qPCR method was chosen due to its high sensitivity and specificity, which allows for precise quantification of the expression level of individual genes. It should be emphasized that this study focused on the analysis of the expression of selected genes, not the entire genome.

### 4.7. Cytotoxicity Analysis

Cytotoxicity assessment was performed using the 2,3-bis-(2-methoxy-4-nitro-5-sulfophenyl)-2H-tetrazolium-5-carboxanilide (XTT) colorimetric assay (Thermo Scientific). The assay is designed to assess cell metabolic activity, which indicates viability, proliferation, and cytotoxicity. All experiments were performed in triplicate. Schwann cells were cultured in a 12-well plate (1 × 10^4^/well) for 10 days in 1.5 mL of complete SCM medium (ScienCell) (the medium was changed every few days). The samples were exposed to the appropriate field: PEMF (100 or 1000 Hz), LIPUS (20 or 40 kHz), PEMF (100 Hz) combined with LIPUS (20 kHz), PEMF (1000 Hz) combined with LIPUS (20 kHz), PEMF (100 Hz) combined with LIPUS (40 kHz), and PEMF (1000 Hz) combined with LIPUS (40 kHz). Positive controls were cells incubated with 100% DMSO (, while negative controls were cultured only in complete SCM medium (ScienCell). Then, XTT/PMS mixtures were added to each well according to the manufacturer’s protocol. After 2 h of incubation in a 5% CO_2_ incubator at 37 °C, the absorbance of the sample was measured using a Synergy HT spectrophotometer (BioTek, Shoreline, WA, USA) at 450 nm.

### 4.8. Genotoxicity Analysis

The comet test (alkaline version) was used to assess the genotoxicity of PEMF and LIPUS fields on Schwann cells. All experiments were performed in triplicate. Schwann cells were cultured at a density of 1 × 104 in 6-well plates in 2 mL of SCM medium (ScienCell). Then, Schwann cells were exposed to the following fields for 10 days: PEMF (100 Hz) combined with LIPUS (20 kHz), PEMF (1000 Hz) combined with LIPUS (20 kHz), PEMF (100 Hz) combined with LIPUS (40 kHz), and PEMF (1000 Hz) combined with LIPUS (40 kHz). Cells treated with 5% DMSO (Sigma-Aldrich, St. Louis, MO, USA) were used as a positive control, and cells cultured only in complete SCM growth medium were used as a negative control. The cells were then suspended in 0.37% low-melting-point agarose that had been precoated with normal melting point agarose (Sigma-Aldrich). The samples were treated with pH 10 buffer for 24 h at 4 °C (2.5 M NaCl, 10 mM Tris, 100 mM EDTA, 1% TritonX-100) (Sigma-Aldrich Corp.). The slides were then incubated for 20 min at 4 °C in developing buffer (300 mM NaOH, 1 mM EDTA) and electrophoresed in electrophoresis buffer (30 mM NaOH, 1 mM EDTA) for 20 min at 4 °C (32 mA, 17 V). After the electrophoresis process, the slides were washed three times with distilled water and allowed to dry at room temperature. The samples were then labelled with the fluorescent substance DAPI. DNA damage to the cells was assessed using a fluorescence microscope to measure the percentage of DNA in the comet tail.

### 4.9. Data Availability

All data supporting the obtained results are available from the corresponding author on reasonable request.

### 4.10. Statistical Analysis

Statistical analyses were performed using Graph Pad Prism software (version 10.1.0). Gene expression analysis data were presented as the mean ± SEM (standard error of the mean) of the experiments performed. In the expression analysis, the distribution of variables was assessed using the Shapiro–Wilk test, and statistical analysis of differences between data groups was performed using the Mann–Whitney U test (for non-normal distribution). Values of *p* < 0.05 were considered statistically significant (*p* * ≤ 0.05, *p* ** ≤ 0.01, *** *p* < 0.001 and *p* **** ≤ 0.0001).

## 5. Conclusions

Our studies provide valuable information on the effect of PEMF and LIPUS therapy on gene expression and Schwann cell function in the context of peripheral nerve regeneration. The results indicate that both therapies have the potential to support regenerative processes through different mechanisms of action. PEMF therapy significantly accelerates Schwann cell proliferation and differentiation and supports remyelination, which is confirmed by the results of increased expression of neurotrophins (BDNF, NGF) and proliferative markers (Ki67). In turn, LIPUS promotes Schwann cell migration and increases the expression of anti-inflammatory cytokines (IL-10), which may contribute to reducing inflammation and supporting peripheral nerve regeneration.

One of the key findings is the synergistic effect of combining PEMF and LIPUS, which enhanced the effect of both therapies in reducing the expression of inflammatory markers (TNF-α, IL-1β, IL-6) and increasing the expression of growth factors and anti-inflammatory cytokines (IL-10, TGF-β). Our studies also show the promising potential of this therapy in modulating the expression of heat shock proteins (CRYABs) and proteoglycans (CSPGs), which is important for improving the regenerative environment.

In summary, PEMF and LIPUS therapies, both individually and in combination, offer promising approaches to support peripheral nerve regeneration. Our results suggest that optimization of therapy parameters can significantly affect their clinical effectiveness, which opens new possibilities for therapies in regenerative medicine.

Future research should focus on several key areas to fully exploit the potential of PEMF and LIPUS therapy in peripheral nerve regeneration. First, it is important to further explore the molecular mechanisms underlying the synergistic effects of both therapies. Understanding the precise signaling pathways and their impact on gene and protein expression in Schwann cells may lead to better adjustment of therapy parameters.

Second, future studies should focus on optimizing the exposure times and intensities of both therapies to maximize their clinical effectiveness. Precise dosing and sequencing of treatments can lead to more individualized and effective treatment plans.

Third, it will be important to conduct in vivo and clinical studies to assess the effects of PEMF and LIPUS on nerve regeneration in more complex models and in clinical settings. These studies should consider the long-term effects of the treatments, their impact on nerve function, and potential side effects.

Finally, studies on the interactions of these therapies with other forms of treatment and their application in different clinical contexts can contribute to the development of more comprehensive and effective therapeutic strategies for the treatment of peripheral nerve injuries.

Despite the valuable insights provided by this research, several additional aspects warrant further investigation to deepen our understanding of the findings. The exclusive use of in vitro models, while enabling a precise analysis of early molecular responses in Schwann cells, does not fully replicate the complexity of in vivo conditions, including the influence of the microenvironment and intercellular interactions critical to nerve regeneration. Additionally, the reliance on qPCR as the primary method of gene expression analysis provides detailed information on molecular changes but requires further corroboration through broader functional studies and protein-level analyses. To validate our findings and enhance their translational potential, future studies should prioritize in vivo experiments using animal models to better mimic real-life conditions and assess long-term therapeutic outcomes. Independent investigations by other research groups will also be essential in confirming these results and exploring their generalizability. Addressing these limitations will strengthen the foundation for clinical applications of PEMF and LIPUS therapies in peripheral nerve regeneration.

## Data Availability

Research data are available from the corresponding author.
